# Effects of an Early Home Visiting Program on Maternal Depression

**DOI:** 10.1007/s10488-025-01440-3

**Published:** 2025-04-04

**Authors:** Kirsten McLaughlin, Regina M. Fasano, Mary Dozier

**Affiliations:** Department of Psychological and Brain Sciences, University of Delaware, Newark, DE, USA

**Keywords:** Maternal depression, Parenting, Early intervention, Risk, Community sample

## Abstract

Maternal depression has been associated with negative parenting behaviors and poor developmental outcomes in children. Home visiting programs have positively impacted parenting behaviors and child outcomes; however, such programs often require specialized, highly trained professionals, resulting in a limited number of home visiting providers. One home visiting parenting program, Attachment and Biobehavioral Catch-up (ABC), does not have requirements regarding experience or background to become an ABC parent coach and deliver the intervention. ABC consists of ten 1-hour weekly sessions for parents of children between 0 and 6 months (ABC-Newborn), 6–24 months (ABC-Infant) or 24–48 months (ABC-Early Childhood). ABC has demonstrated efficacy in improving parental sensitivity and children’s developmental outcomes. A randomized clinical trial in one community implementation setting showed that ABC decreased maternal depressive symptoms. The current study aimed to replicate this finding across multiple implementation sites and expand on it by exploring if the effect differed by ABC model. Data included a community sample of 163 families from six countries who completed ABC. Maternal reports of depressive symptoms were collected prior to and after receiving ABC. Results showed a significant decrease in maternal depressive symptoms scores from pre-intervention to post-intervention regardless of ABC model. Findings demonstrate that a home visiting parenting intervention program can successfully leverage non-traditional mental health providers to ensure that mothers and children receive necessary resources and support.

## Introduction

The increasing prevalence of depression is a major global public health issue. From 1990 to 2017, the number of people with depression worldwide was estimated to increase from 172 million to 258 million (Liu et al., 2020). Moreover, women and children are disproportionately impacted as women are twice as likely as men to develop major depression, with the greatest risk for a major depressive episode occurring during women’s childbearing years (Accortt et al., 2008). A meta-analysis found that maternal depressive symptoms increased the likelihood of negative parenting behaviors including impatience, low sensitivity, hostility and overall negative parent–child interactions (Lovejoy et al., 2000). Despite these effects, depression is widely untreated due to barriers to receiving professional help and a lack of mental health resources. It is therefore imperative that healthcare systems seek innovative solutions to address this global public health issue. One such solution is leveraging community members and non-mental health providers to implement mental health and parenting interventions, such as the Attachment and Biobehavioral Catch-up (ABC), to ensure women and children receive the resources and support that they need.

### Maternal Depression and Child Outcomes

Maternal depression has been associated with poor developmental outcomes in children (Mirhosseini et al., 2015; Slomian et al., 2019). A meta-analysis of maternal depressive symptoms and child cognitive development found that at 6–8 weeks of age, infants with mothers experiencing postpartum depressive symptoms performed worse across cognitive domains (e.g., memory, language development) compared to infants whose mothers did not experience symptoms (Liu et al., 2017). Similar effects were shown in early childhood, as children whose mothers had high depressive symptoms performed worse on cognitive tasks compared to those whose mothers had few to no depressive symptoms (Liu et al., 2017). Further, exposure to maternal depression has been associated with increased socioemotional (e.g., affect, social communication) and behavioral problems (e.g., internalizing and externalizing problems) in two year olds (Junge et al., 2017; Suarez et al., 2023).

At six years of age, children whose mothers had continually high depressive symptoms since the child’s birth were more likely to have more externalizing behavior problems compared to children whose mothers had low levels or no depressive symptoms (Ashman et al., 2008). Researchers have also shown that school-aged children exposed to maternal depression have more difficulties with social competence, emotional maturity (e.g., prosocial behavior, anxiety), and physical health (e.g., gross/fine motor skills) than children who are not exposed to maternal depression (Ashman et al., 2008; Maughan et al., 2007; Wall-Wieler et al., 2020), which was especially true for children whose mothers were depressed during the first year of the child’s life or in the year before the child started school (Maughan et al., 2007; Wall-Wieler et al., 2020). Further, children and adolescents exposed to maternal depression were likely to experience increased internalizing and externalizing symptomatology and increased negative affect (Goodman et al., 2011). The persisting negative effects of exposure to maternal depression in early life highlight the need for early interventions to prevent these outcomes through altering developmental trajectories.

### Home Visiting Parenting Programs

Home visiting parenting programs typically consist of individual parenting support in which trained clinicians implement services in the family’s home (Duffee et al., 2017). Working primarily with pregnant women or families with young children, home visiting parenting programs vary in scope and target. Due to the federal establishment of the Maternal, Infant, and Early Childhood Home Visiting (MIECHV) Program, many high-risk families have participated in home visiting parenting programs over the last decade. In 2022 alone, the MIECHV Program served approximately 138,000 parents and children and provided over 840,000 home visits (Brimsek et al., 2022). Home visiting parenting programs have been shown to have positive effects on children’s developmental, health, and educational outcomes (Avellar & Supplee, 2013; Filene et al., 2013). Additionally, data from randomized controlled trials have shown a significant impact of home visiting on the occurrence of child maltreatment, including fewer reports and substantiations to Child Protective Services (CPS) in home-visited groups compared to controls (Olds et al., 1997; Zielinski et al., 2009).

Current home visiting parenting programs tend to rely on specialized, highly trained professionals who have advanced degrees and undergo years of training. However, this reliance on trained professionals creates an obstacle, as it limits the number of home visiting providers. This shortage of highly trained professionals negatively affects families in need of home visiting services, including mothers experiencing depression and their children. To circumvent this problem, ABC does not require home visiting providers to have a specific academic degree, licensure, or a set number of years of experience to become trained in implementing the ABC program. This allows ABC to leverage non-profit organization staff, public educators, and other community members to ensure that families are receiving the necessary services.

### ABC Intervention

​​ABC is a home visiting parenting program that consists of ten 1-hour weekly sessions for parents of children between 0 and 6 months (ABC-Newborn), 6–24 months (ABC-Infant), or 24–48 months (ABC-Early Childhood) who have experienced early adversity, such as maltreatment (abuse/neglect), involvement in foster care or disruption in caregivers, witnessing intimate partner violence, or time spent in a neonatal intensive care unit (NICU). ABC has been studied and found efficacious in randomized controlled trials with families referred by CPS, foster families, and parents adopting children from international orphanage care (e.g., Bernard et al., 2015; Bernard, Hostinar et al., 2015; Lind et al., 2017, 2020; Yarger et al., 2020). ABC is designed to improve parental sensitivity by focusing on three parenting behaviors identified as essential for children who have experienced early adversity: (1) responding to child distress with *nurturance*, (2) *following the child’s lead with delight* during everyday interactions and play, and (3) *avoiding frightening behaviors* (Dozier & Bernard, 2019). At each weekly session, trained ABC parent coaches discuss these targets with parents using manualized content and frequent in-the-moment comments. For ABC-Newborn, ABC-Infant, and ABC-Early Childhood, manualized content includes showing example videos, citing research support, and encouraging practice activities between parents and their children. All three models also use frequent in-the-moment comments to support target-relevant caregiver behaviors. These in-the-moment comments have been found to increase parental sensitivity and are considered the active ingredient of ABC in promoting sensitive parental behavior (Caron et al., 2018).

To be eligible to train as a parent coach in ABC, potential coaches undergo a brief screening process with ABC staff members. The screening process uses two measures to identify providers who are likely to implement ABC with fidelity. The first measure assesses the person’s valuing of attachment/openness using the Brief Adult Attachment Interview (AAI; George et al., 1985). The second measure uses video vignettes of ABC sessions to assess the person’s ability to make in-the-moment comments (Caron et al., 2018). Each of these measures is scored on a 1–5 scale, with an average score of 3 or higher indicating that a potential coach has passed the screening. Regardless of a person’s work experience or educational background, they are eligible to participate in the screening process, allowing ABC to leverage non-traditional mental health providers such as non-profit organization staff, parents who themselves have gone through the ABC intervention, and other community members to implement the program.

After potential coaches pass the screening, they participate in three half-days of intensive training that introduce ABC, including manualized content and in-the-moment commenting. After this initial training, coaches participate in weekly clinical and fidelity supervision meetings that occur over the course of 6 months. Clinical supervision consists of a small group meeting of two to three parent coaches led by an ABC clinical supervisor. These supervision meetings are used to review session videos and discuss case conceptualization, manual adherence, and logistics. Fidelity supervision consists of an individual meeting led by an ABC fidelity supervisor. During these meetings, parent coaches and their fidelity supervisors review coding of the parent coaches’ in-the-moment commenting and fidelity to the model from session videos (Caron & Dozier, 2019; Meade et al., 2014).

ABC fidelity is based on parent coaches’ commenting rate, percentage of on-target comments, and the average component level of their comments. An on-target comment is when the parent behavior and the parent coach’s comment match (e.g., the parent follows the child’s lead and the parent coach comments on the parent following the lead). The commenting rate should be equal to or greater than one on-target comment per minute. The percentage of on-target comments should be equal to or greater than 80% of all comments. The average component level should be equal to or greater than 1. The number of components quantifies the amount and quality of information in the parent coach’s response. Components either (a) describe the behaviors of parent and child, (b) label the intervention target, or (c) provide an outcome concerning the developmental and/or relational effects the ABC-targeted behavior has on the child.

Randomized clinical trials have shown the efficacy of ABC in improving parental sensitivity (Bick & Dozier, 2013; Yarger et al., 2020), promoting secure attachment (Bernard et al., 2012) and child self-regulation (Lind et al., 2017), and normalizing patterns of diurnal cortisol production, an indicator of physiological regulation (Bernard et al., 2015; Bernard, Hostinar et al., 2015). Across these trials, improved parental sensitivity has emerged as an empirically tested mechanism of change for a number of the positive child outcomes (e.g., Garnett et al., 2020; Raby et al., 2019). Given that parental sensitivity is conceptualized as the intervention mechanism, it is the primary outcome assessed in community implementation settings. When assessing pre-ABC to post-ABC parental sensitivity change in community implementation settings, researchers have found that the effect size for this improvement is large, and similar to what has been seen in previous clinical trials (Perrone et al., 2021; Roben et al., 2017). A randomized clinical trial in one community implementation setting showed that ABC had a significant effect on decreasing maternal depressive symptoms (Perrone et al., 2021). This was notable as maternal depression is not directly addressed through ABC. The current study aimed to replicate this finding across multiple implementation sites and expand on it by exploring if the effect differed by the ABC model (ABC-Newborn, ABC-Infant, ABC-Early Childhood) that mothers received.

### The Current Study

Given the prevalence of maternal depression, its impact on child development, and the need to leverage non-traditional mental health professionals, the current study assessed pre-intervention to post-intervention change in maternal depressive symptoms in community implementation settings. We also examined whether this change differed by ABC model. We hypothesized that maternal depressive symptoms would decrease from pre-intervention to post-intervention. Given that the models share significant overlap in content and all use in-the-moment commenting to address parent behaviors, we did not expect that change in maternal depressive symptoms would depend on which ABC model mothers received.

## Method

### Participants

A total of 381 families completed ABC from March 2021 through November 2023. Of the 381 families, 163 (43%) completed the voluntary pre-intervention and post-intervention assessments. Families were referred to parent coaches by agencies to receive ABC for various reasons (e.g., child maltreatment, involvement in foster care, etc.).

### Agencies

The agencies that parent coaches worked for varied in terms of their geographic location, organizational structure, geographic reach, and services that they provided. Agencies spanned across the following six countries: Australia, Canada, South Korea, Sweden, the United Kingdom, and the United States. Within the United States, agencies were located in the District of Columbia and the following 13 states: California, Kansas, Maryland, Minnesota, Missouri, North Carolina, North Dakota, New York, Oklahoma, Pennsylvania, South Carolina, Utah, and Washington. As for organizational structure, agencies consisted of nonprofit agencies, for-profit agencies, academic institutions, government agencies, and community-based organizations. In terms of geographic reach, some agencies focused on local/regional outreach and others implemented services on a state/national level. Agencies also varied in the services that they provided ranging from mental health and developmental support to early childhood services to counseling and therapy to community/social support.

### Procedures

Prior to completing the first session of ABC and following the completion of the last session of ABC, parents completed a series of demographic and psychosocial questionnaires.

#### Maternal depressive symptoms

Maternal depressive symptoms were calculated from parents’ responses on the Center for Epidemiologic Studies Depression Scale (CES-D; Radloff, 1977) during the pre- and post-intervention assessments. This 20-item form asks respondents to indicate the frequency of depressive symptoms during the past week on a 4-point Likert Scale (“0 = Rarely or none of the time” to “3 = Most or all of the time”). The total score is created by reverse coding four items related to positive feelings and then summing all 20 items, with a higher score indicating a higher frequency of depressive symptoms. The sample showed excellent internal consistency for maternal depressive symptoms (Cronbach’s alpha = 0.93).

### Analytic Plan

A one-way analysis of variance (ANOVA) was first conducted to see if there was a significant difference in pre-intervention maternal depressive symptom scores between ABC models. A repeated measures ANOVA was then conducted to examine main effects of time (pre/post) and ABC model (ABC-Newborn, ABC-Infant, ABC-Early Childhood), and the interaction of time and ABC model on maternal depressive symptom scores. All analyses were conducted using SPSS Version 6.13.

## Results

### Descriptive Statistics

All caregivers identified as women (*N* = 163) and 81% identified as being the birth parent of the child participating in ABC. The remaining 19% of caregivers identified as either a foster parent, adoptive parent, or kinship relation of the child participating in ABC. Average caregiver age in years was 33.67 (*SD* = 9.14). Regarding primary caregiver race, 58% of caregivers identified as White, 17% identified as Black or African American, 5% identified as Asian, 2% identified as Middle Eastern or Northern African, 1% identified as American Indian, Alaskan Native, Indigenous or First Nations, 1% identified as Other, and 15% chose not to identify. Of the 161 caregivers who completed information regarding their ethnicity and family’s welfare status, 21% identified as having Hispanic, Latina, or Spanish origin and 29% identified as currently being on welfare. Of the 162 caregivers who completed information regarding caregiver education level, 10% completed some high school, 32% completed high school or a GED, 17% completed some undergraduate education, 25% completed an undergraduate program, 6% completed some master’s level education, 7% completed a master’s program, and 4% completed some or all of a doctoral program. Child’s biological sex at birth was reported to be female for 51% of the children whose families participated in the assessments. Thirteen families participated in ABC-Newborn, 104 participated in ABC-Infant, and 46 participated in ABC-Early Childhood. Descriptive statistics for participants by ABC model are presented in Table 1.

**Table 1:** Demographic Statistics for Participants

			All ABC Models				ABC-Newborn				ABC-Infant				ABC-Early Childhood	
	*N*	%	Mean (SD)	Range	*N*	%	Mean (SD)	Range	*N*	%	Mean (SD)	Range	*N*	%	Mean (SD)	Range
*Caregiver Demographics*																
Caregiver age (in years)	161		33.67(9.14)	18.00– 67.00	13		31.85(8.33)	22.00– 5.00	102		33.21(9.83)	18.00–67.00	46		35.22(7.90)	20.00–52.00
Caregiver sex	163				13				104				46			
Male	0	0			0	0			0	0			0	0		
Female	163	100			13	100			104	100			46	100		
Relationship to Child	163				13				104				46			
Birth parent	132	81			13	100			85	82			34	74		
Foster parent/adoptive parent/kinship relation	31	19			0	0			19	18			12	26		
Caregiver Race	163				13				104				46			
White	95	58			11	85			66	63			18	39		
Black or African American	28	17			0	0			17	16			11	24		
Asian	8	5			0	0			5	5			3	7		
Middle Eastern or Northern African	3	2			1	8			1	1			1	2		
American Indian, Alaskan Native, Indigenous, or First Nations	2	1			0	0			1	1			1	2		
Other	2	1			0	0			1	1			1	2		
Chose not to identify	25	15			1	8			13	13			11	24		
Hispanic, Latina, or Spanish Origin	161				13				102				46			
Yes	34	21			0	0			16	16			18	39		
No	127	79			13	100			86	84			28	61		
Caregiver Education Level	162				13				103				46			
Some high school	16	10			2	15			10	10			4	9		
High school diploma or GED	52	32			4	302			30	29			18	39		
Some Undergraduate level education	27	17			2	15			20	19			5	11		
Undergraduate degree	40	25			1	8			26	24			13	28		
Some Master’s level education	9	6			1	8			5	5			3	7		
Master’s degree	12	7			2	15			7	7			3	7		
Some doctoral level education or a doctoral degree	6	4			1	8			5	5			0	0		
Family Welfare Status	161				13				102				46			
Receiving Welfare	45	29			2				28	27			15	33		
*Child Demographics*																
Child age (in months)	161		16.30 (12.00)	1– 48.00	13		2.08(1.12)	1.00–4.00	103		11.58(6.08)	1.00–27.00	43		31.88(9.26)	2.00–47.00
Child sex	163				13				103				46			
Female	83	51			5	38			58	56			20	43		
Male	80	49			8	62			45	44			26	57		

**Fig. 1:**
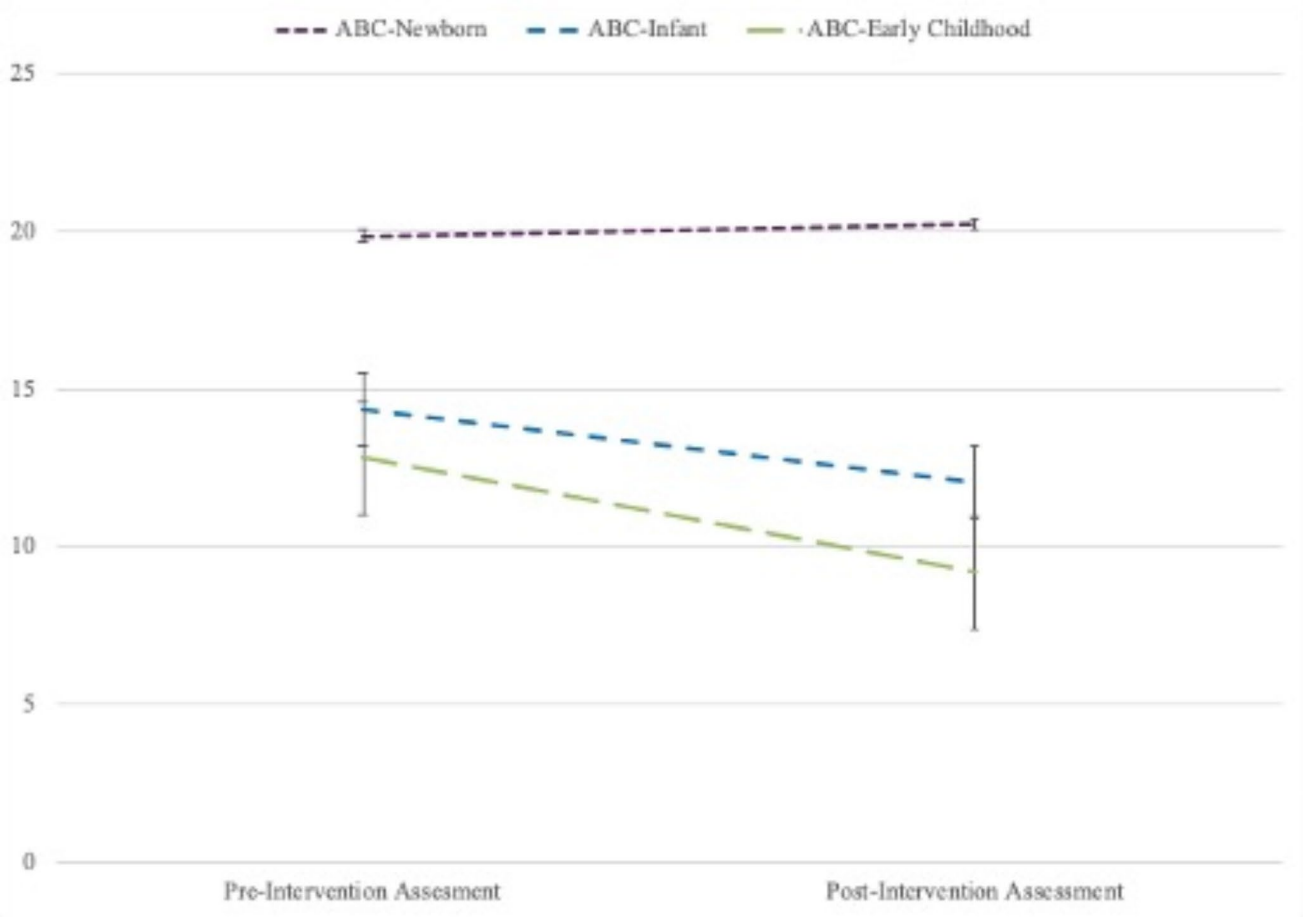
Means of Maternal Depressive Symptoms Scores at Pre- and Post-Intervention by ABC Model. *Note*. The figure shows a profile plot for the estimated marginal means and standard errors of maternal depressive symptoms scores at the pre- and post-intervention assessments by ABC model. The average depressive symptoms score for mothers who received ABC-Newborn at pre-intervention was 19.85 (*SD*=9.16) and 20.23 (*SD*=14.18) at post-intervention. The average depressive symptoms score for mothers who received ABC-Infant was 14.12 (*SD*=11.51) at pre-intervention and 11.89 (*SD*=10.92) at post-intervention. The average depressive symptoms score for mothers who received ABC-Early Childhood was 12.76 (*SD*=11.13) and 9.28 (*SD*=9.42) at post-intervention.

Maternal pre-intervention depressive symptom scores were not significantly correlated with any caregiver or child demographic factors. Across ABC models, maternal depressive symptom scores ranged from 0 to 53 at the pre-intervention and from 0 to 52 at the post-intervention, with 41% (*n* = 66) of mothers scoring at or above the clinical cutoff of 16 at the pre-intervention and 32% (*n* = 52) scoring at or above the clinical cutoff at post-intervention. A paired samples t-test showed that there was a significant change in maternal depressive symptom scores for mothers who scored at or above the clinical cut off at the pre-assessment from pre-assessment (M = 25.78, SD = 8.19) to post-assessment (M = 19.97, SD = 11.48, t(65) = 4.63, *p* <.001). Means of pre- and post-intervention assessment maternal depressive symptoms scores by ABC model are shown in Fig. 1.

### Results from ANOVAs

The one-way ANOVA showed that there was no significant difference in pre-intervention maternal depressive symptom scores between ABC models (F(2,160) = 1.987, *p* =.141, η_p_^2^ = 0.02). The repeated measures ANOVA showed that the main effect of time was significant (*F*(1, 160) = 4.15, *p* =.043, η_p_^2^ = 0.03), such that depressive symptom scores decreased from the pre-intervention assessment to the post-intervention assessment. There also was a significant main effect of ABC model on maternal depressive symptom scores (*F*(2) = 3.97, *p* =.021, η_p_^2^ = 0.71). Post-hoc comparisons using the Bonferroni adjustment for multiple comparisons indicated that post-intervention depressive symptom scores for mothers who received ABC-Newborn were 9.04 points higher than the depressive symptom scores for mothers who received ABC-Early Childhood (*p* =.017). There was no significant difference in post-intervention depressive symptom scores between mothers who received ABC-Newborn and mothers who received ABC-Infant (*p* =.073), or between mothers who received ABC-Infant and mothers who received ABC-Early Childhood (*p* =.683). The interaction effect of time and ABC model was not significant (*F*(2,160) = 1.23, *p* =.297, η_p_^2^ = 0.02).

## Discussion

The current findings expand on previous research, showing the effectiveness of ABC in reducing maternal depressive symptoms over the course of its 10-week program in community implementation settings. This finding is especially critical as the prevalence of maternal depression continues to rise, which can negatively impact children’s cognitive, socioemotional, and behavioral development (Haight et al., 2019; Mirhosseini et al., 2015; Slomian et al., 2019). Notably, this finding demonstrates that ABC, a home visiting program that can be delivered by laypersons, is effective in decreasing maternal depressive symptoms over a relatively short period of time. This leverage of non-traditional mental health providers provides additional support for families who need home visiting services, for the betterment of mothers and their children.

### Potential Implications of ABC on Maternal Depressive Symptoms

ABC focuses on three target parenting behaviors (i.e., nurturance, following the child’s lead with delight, and avoiding frightening behaviors) to improve parental sensitivity (Dozier & Bernard, 2019). Although ABC does not directly target maternal depressive symptoms, the current findings indicate that these symptoms are nonetheless impacted. While outside the scope of the current study, there are several possible explanations for these findings that should be further explored. Other factors that were not measured in the current study (e.g., parental sensitivity, parental self-efficacy, etc.) should be incorporated in future research to fully elucidate our understanding of ABC’s effects on maternal depressive symptoms.

It is possible that the decrease in maternal depressive symptoms may be attributable to an increase in parental sensitivity. Parental sensitivity has been shown to be disrupted among depressed mothers (Campbell et al., 2007; Dix & Yan, 2014; Kaplan et al., 2015) and ABC has been shown to enhance parental sensitivity (Bick & Dozier, 2013; Perrone et al., 2021; Roben et al., 2017; Yarger et al., 2020). It is also possible that a bidirectional relationship exists between maternal depressive symptoms and parental sensitivity. For example, it may be that depressive symptoms negatively impact parental sensitivity, which may in turn exacerbate the parent’s depressive symptoms. If true, then decreasing depressive symptoms could improve parental sensitivity, further decreasing depressive symptoms. Follow-up studies should aim to examine the relationship between parental sensitivity and maternal depressive symptoms for families receiving ABC. Another possibility is that there are underlying mechanisms, such as parental self-efficacy, driving the association between maternal depressive symptoms and parental sensitivity.

Researchers have shown that parental self-efficacy is inversely related to parental depression (Haslam et al., 2006; Leahy-Warren et al., 2012; Teti & Gelfand, 1991), and that parents with higher self-efficacy are better at adapting, coping, and persisting when facing challenges (Bandura, 1982; Coleman & Karraker, 2000; Teti & Gelfand, 1991). Parent coaches may increase parental self-efficacy by providing parents with strategies and in-the-moment comments aimed at increasing parental sensitivity and forming secure parent-child attachments. In this way, ABC may increase parents’ sense of self-efficacy regarding their own parenting and decrease maternal depressive symptoms. Further research is needed to examine this potential mechanism in regard to ABC and maternal depressive symptoms.

Other third variables, such as maternal perceived social support, may directly drive the association between ABC and maternal depressive symptoms. Social support has been shown to play a key role in postpartum depression, such that parents who receive more social support are less likely to experience maternal depression (Ege et al., 2008; Gao et al., 2009; Leahy-Warren et al., 2012; O’Hara & Swain, 2009). For some mothers, their parent coach may be their only source of social support. Thus, it is plausible that ABC may indirectly influence maternal depression by providing parents with increased social support. Future research should incorporate measures of perceived social support at the pre- and post-intervention assessments of ABC to fully elucidate the association between perceived social support and maternal depressive symptoms.

### Effects by ABC Model

The average depressive symptoms scores at pre- and post-intervention for mothers who received ABC-Newborn were above the clinical cutoff of 16. These scores are consistent with well-established research that maternal depressive symptoms are most likely to occur, and are most severe, during pregnancy and postpartum periods (Banti et al., 2011; Evans et al., 2001; França & McManus, 2018; Kothari et al., 2016). Additionally, these findings may be due to the fact that the current sample only included 13 mothers who received ABC-Newborn, compared to the 150 mothers who received ABC-Infant or ABC-Early Childhood. Thus, there may not have been enough cases to fully examine the effects of ABC-Newborn on maternal depressive symptoms. Findings should be replicated in a larger sample that contains an equal number of mothers per ABC model.

### Strengths and Limitations

The current observational study demonstrates the efficacy of ABC and its role in decreasing maternal depressive symptoms in community implementation settings using non-traditional mental health professionals. A strength of the study is that the findings are widely generalizable due to the size and diversity of the sample regarding geographical location, maternal race and ethnicity, and maternal education level. Another strength of the current study is that it is the first to examine the effects of ABC on maternal depressive symptoms in community implementation settings. A previous randomized clinical trial demonstrated the efficacy of ABC in decreasing maternal depressive symptoms (Perrone et al., 2021). However, the finding had not yet been examined through observational assessment in community implementation settings. Testing the efficacy in community settings is vital, as many promising interventions are effective in randomized controlled trials, but few show such efficacy when implemented in community settings (Glasgow et al., 2003; Weisz et al., 1992).

Despite these strengths, care should be taken in interpreting these findings due to several limitations. One such limitation is the reliance on maternal report for measuring maternal depressive symptoms. Self-reported measures are subject to social desirability response bias, which is the tendency to underreport socially undesirable behaviors and overreport desirable ones. This bias may lead mothers to underreport their levels of depressive symptoms. To avoid social desirability response bias, ABC could train parent coaches to implement a structured clinical interview, such as the Mini International Neuropsychiatric Interview or the Structured Clinical Interview for DSM-5 (First et al., 2015; Sheehan et al., 1998). This would provide researchers with a more objective measure of maternal depressive symptom scores.

Additionally, the current study did not account for other intervention services or therapies that the family may have been receiving in tandem with ABC. Some of the families that participated in ABC were referred through state health departments and may have been participating in other programs simultaneously with ABC. While policies may have differed due to geographical location, the implementation of ABC was consistent across locations due to the standardized training process that parent coaches underwent. Further, by training community members as ABC parent coaches, ABC was implemented with families in their primary language. Lastly, the small number of mothers who received ABC-Newborn may have limited our ability to fully test whether changes in maternal depressive symptom scores differ by the ABC model that families received. Future work should include a more balanced sample across models to fully elucidate our understanding of whether changes in maternal depressive symptoms scores differ by the ABC model that families receive. To do so, ABC needs to ensure that it is working with agencies that are actively receiving referrals for parents of children between 0 and 6 months.

### Practice and Policy Implications

The findings that ABC decreases maternal depressive symptoms underscore the transformative potential of integrating non-traditional mental health providers into home visiting programs. This approach not only enhances the accessibility and effectiveness of home visiting programs but also aligns with the growing recognition of the value of diverse service delivery models in addressing complex public health issues. To ensure the sustainability of such programs, policymakers should advocate for the inclusion of non-traditional mental health providers in home visiting programs and there should be dedicated funding streams and reimbursement mechanisms that support the employment and training of these providers. Additionally, training and certification programs for these non-traditional providers should be developed. ABC provides a clear model for a standardized training and certification process that other home visiting programs should strive to replicate. These comprehensive training programs are essential for equipping non-traditional mental health providers with the necessary skills and knowledge needed to effectively deliver services. Integrating non-traditional mental health providers into home visiting programs like ABC represents a significant step toward improving mental health outcomes for mothers and children, ultimately fostering a more supportive and resilient healthcare system.

## Conclusion

Given the increasing prevalence of maternal depression and lack of mental health resources and barriers to receiving professional help, it is crucial that healthcare systems seek innovative solutions to address this global public health issue. ABC provides an example of how home visiting parenting intervention programs can leverage non-traditional mental health providers to ensure that mothers and children are receiving necessary resources and support. ABC allows any person, regardless of experience or background, the opportunity to become an ABC parent coach if they pass screening criteria. When the need for advanced degrees and training is taken out of the equation, more community members can be trained in interventions that help families by providing support for parents, which in turn enhance children’s developmental outcomes.
